# High Effective Preparation of Amorphous-Like Si Nanoparticles Using Spark Erosion Followed by Bead Milling

**DOI:** 10.3390/nano11030594

**Published:** 2021-02-27

**Authors:** Mingcai Zhao, Juan Zhang, Wei Wang, Qi Zhang

**Affiliations:** 1 College of Mechanical and Electrical Engineering, Nanjing University of Aeronautics and Astronautics, Nanjing 210016, China; mingcaizhao@nuaa.edu.cn (M.Z.); zhangjuan5433@126.com (J.Z.); 2 BCMaterials, Basque Center for Materials, Applications and Nanostructures, UPV/EHU Science Park, 48940 Leioa, Spain; 3 IKERBASQUE, Basque Foundation for Science, Plaza Euskadi, 5, 48009 Bilbao, Spain; 4 State Key Laboratory of Advanced Technology for Materials Synthesis and Processing, School of Materials Science and Engineering, Wuhan University of Technology, Wuhan 430070, China

**Keywords:** Silicon nanoparticle, amorphous-like, nanocrystal, spark erosion, bead milling

## Abstract

This work aims to prepare the silicon nanoparticles with the nanocrystal-embedded amorphous structure through spark erosion followed by bead milling. Spark erosion breaks up monocrystal silicon ingots into micro/nanoparticles, refines the crystal grains, makes the crystals randomly disordered, and increases isotropic character. Bead milling further refines the crystal grains to a few nanometers and increases the amorphous portion in the structure, eventually forming an amorphous structure with the nanocrystals embedded. Spark erosion saves much time and energy for bead milling. The crystallite size and the amount of amorphous phase could be controlled through varying pulse durations of spark discharge and bead milling time. The final particles could contain the nanocrystals as small as 4 nm and the content of amorphous phase as high as 84% and could be considered as amorphous-like Si nanoparticles. This processing route for Si nanoparticles greatly reduced the production time and the energy consumption and, more importantly, is structure-controllable and scalable for mass production of the products with higher purity.

## 1. Introduction

Silicon nanomaterials have specific physical and chemical characters due to their small size and large surface area, which find them a broad application in many fields [1]. In the medical field, Erogbogbo et al. used silicon quantum dots with nanocrystalline structures as luminescent labels to image cancer cells [2]. Owing to superior optical properties, Si nanostructures were applied in biosensing and used as contrast agents [3,4]. In addition, drugs could be efficiently delivered by special Si nanostructures on special occasions rather than traditional oral and injection [5]. In the photovoltaic field, nanosized silicon ink could improve solar cell efficiency obviously with proper technology [6]. In the microelectronic field, crystalline Si nanoparticles present good electroluminescence and provide a higher light-emission efficiency than bulk Si [7]. In the new energy field, the application to the lithium-ion battery (LIB) has pushed the study of Si nanomaterials to the highest level, and Si is considered to be the best alternative LIB anode material [8,9,10,11,12,13].

Apart from the particle size and the exterior structures of Si materials, scientists gradually pay more attention to the internal structures (nanocrystals and amorphous regions), as internal structures also have an effect on their physical and chemical properties [14]. Crystalline silicon is anisotropic, while amorphous silicon is isotropic [15]. Both structures have different application scenarios; for example, nanocrystal could be used to map cells because of its luminescent property. Amorphous structures have better kinetics and fracture properties than crystals during lithiation, and Li-ions have a faster diffusion rate in amorphous silicon than in crystal silicon. Amorphous Si spheres with a diameter of up to 870 nm will not crack during the lithiation process, while the size of crystalline one is 150 nm [16,17,18]. As a result, it is an important issue to research Si nanomaterials with nanocrystals/amorphous structures and how to control the nanocrystal size and the amorphous phase content.

Si nanoparticles prepared by chemical methods, such as ultrahigh vacuum chemical vapor deposition (CVD) [19], plasma synthesis method [20], magnesiothermic reduction method [21], are usually highly purified and have a uniform size distribution. However, the chemical reaction methods always need harsh reaction conditions, complicated processing routes, poor controllability, high cost, low yield, etc., which seriously restrict their large-scale manufacturing. On the other hand, the average particle size of Si nanoparticles prepared by physical methods, such as laser ablation [22], ultrahigh vacuum method [23], spark erosion [24], could be several nanometers with particular parameters, but these methods hardly avoid the existence of microsized particles and the nanoparticles always aggregate and coalesce to form big particles [25]. High-energy ball milling and bead milling are frequently used methods to prepare sub-micron and nanomaterials. However, producing nanoparticles by high-energy ball milling or bead milling requires the particle size of raw materials below a few microns when the size of balls or beads is small and even up to 0.1 mm. Therefore, the raw bulk materials, for example, Si ingots, must be crushed many times by many other methods before the use of high-energy ball milling or bead milling, which usually results in tedious and energy-consuming processing. Long-time ball milling and bead milling will also reduce the purity of Si particles because of the wear loss of balls or beads [26,27,28]. In addition, the inner structure of Si micro/nanoparticle is different and easier to form amorphous regions because these particles are vaporized or melted and cooled during the spark erosion process.

This paper presents a combined process route to prepare Si nanoparticles using spark erosion followed by bead milling. Si micro/nanoparticles, as the precursor materials for bead milling, are prepared from the monocrystal silicon ingots directly by spark erosion which is a short and energy-saving process. The smaller size of precursor materials shortens the bead milling time to achieve nanosized Si particles. More importantly, the inner structure of prepared Si micro/nanoparticles is completely different from those refined by other crushing methods because, during the spark erosion process, Si particles undergo a process from amorphization at high-temperature to recrystallization at rapidly cooling. The repeated impacts in the bead milling process further refine the grains and generate amorphous structures, eventually forming an amorphous-like structure with a small number of nanocrystals embedded inside the amorphous matrix. The morphology and internal structure of prepared Si particles are analyzed, respectively, and the amorphization degrees are estimated by fitting Raman spectra and XRD patterns, respectively.

## 2. Materials and Methods

### 2.1. Spark Erosion

**Figure 1:**
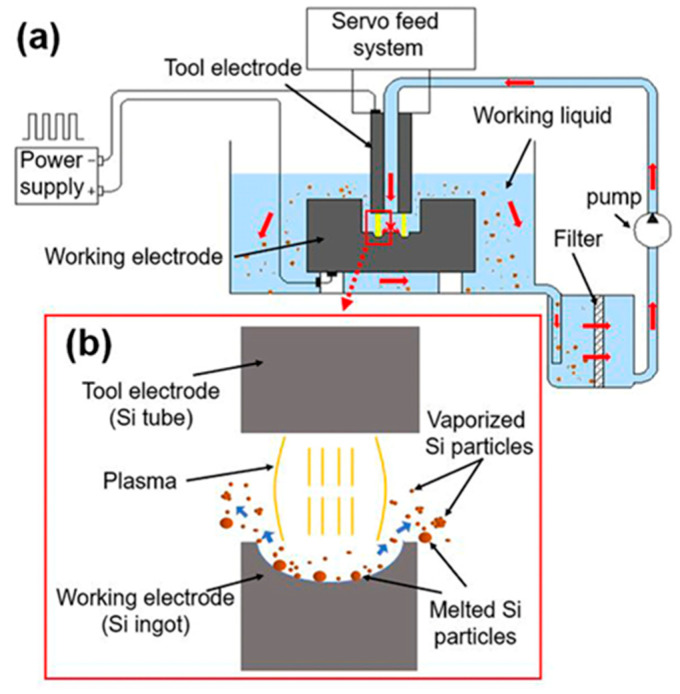
(**a**) Schematic view of spark erosion system and (**b**) illustration of the working area.

The system used for spark erosion (shown schematically in Figure 1a) consists of a pulsed DC power supply, an automated servo system, tool electrode (Si tube), working electrode (Si ingot), working fluid (deionized water), a high-pressure pump for rapid dielectric flushing, a filter for siphoning debris particles, and a container for collection.

The use of doped electrodes would increase the mass production rate, and Zhang et al. needed to purify debris because they used copper tool electrodes [24,29]. Hereby, both working electrode and tool electrodes are prepared by using a sort of boron-doped *P*-type monocrystal silicon material (resistivity is 0.01 Ω·cm^−1^). As shown in Figure 1b, the electrode material in the spark processing is melted and gasified to form nanoscaled particles agglomerated by the vaporized silicon atom clusters and melted microscale particles [30]. When the radius of the equivalent hot spot is used to approximately calculate the radius of the plasma, pulse duration has a far greater impact on power density than other parameters [24,31]. Therefore, two different pulse durations are selected in the experimental work for comparison. The long pulse duration is 200 μs, and the obtained particles are referred to as Si_L_, while the short pulse duration is 1 μs, and the obtained particles are referred to as Si_S_. The operating conditions are shown in Table 1.

### 2.2. Bead Milling

**Figure 2:**
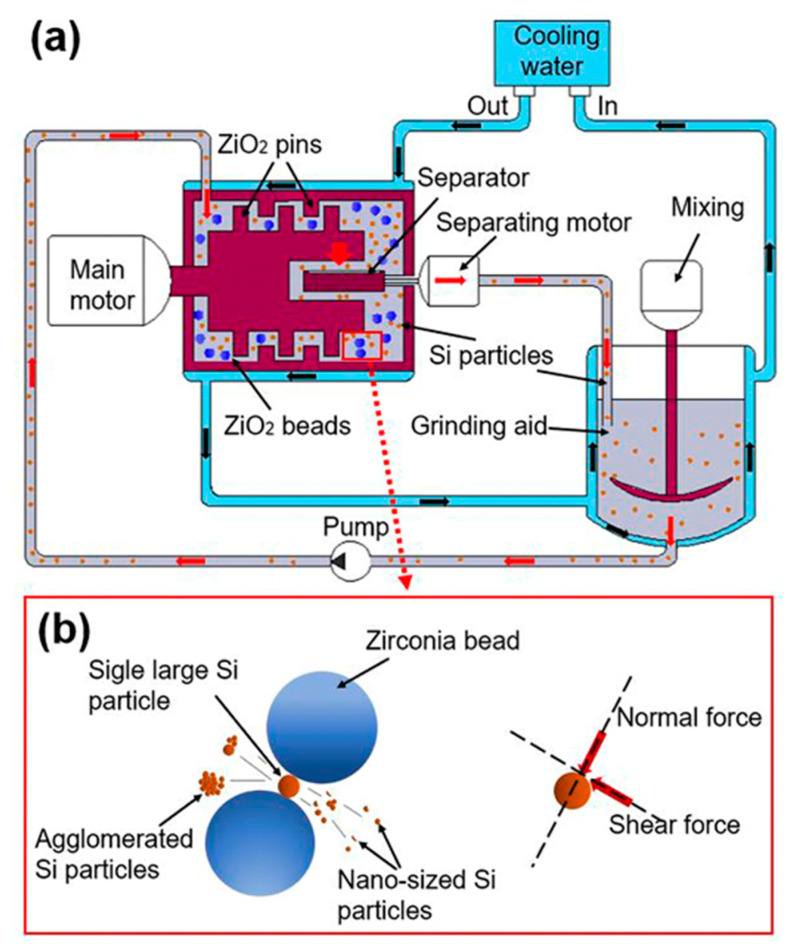
(**a**) Schematic view of bead milling system and (**b**) illustration of the working area.

As shown in Figure 2a, the bead milling system consists of an external circulating cooling water system, material crushing, including main motor, separating motor, separator, mixing, ZrO_2_ pins, ZrO_2_ beads and pump. The process of particle refinement is shown in Figure 2b. During the collision of beads with high mechanical energy, normal and shear forces are the main forces to break particles [32,33]. During the crushing process, agglomerated Si particles were opened, and single large Si particles were ruptured into nanosized particles.

The state of the Si micro/nanoparticles prepared by spark erosion will affect the results after bead milling [27,28]. The micro/nanoparticles Si_L_ and Si_S_ prepared with long and short pulse durations were bead milled for 8 h with the same parameters to obtain nanoparticles Si_L+M_ and Si_S+M_. At the same time, in order to verify the effect of bead milling time parameters on the degree of amorphization, a set of control experiments for Si_S_ bead milling for 12 h was added to obtain Si_S+MM_. The operation conditions are listed in Table 2.

### 2.3. Characterization

A Malvern NanoZS laser particle size analyzer (LPSA, Malvern, UK), the measurement range, of which is 10–10,000 nm, was used to measure and analyze the average size and distribution of particles. A scanning electron microscope (SEM) from Hitachi (S-4800, Tokyo, Japan) was used to observe the microstructure and surface morphology of particles. X-ray diffraction (XRD) from Japanese Science Company (D/max2500PC, Tokyo, Japan) was used for the determination of the microstructure of the particles. Radiation CuKα (λ = 0.15406 nm), working current 100 mA, working voltage 40 KV, scanning range 2θ = 10–90° at a scanning rate of 6 °/min are selected for the tests. The transmission electron microscope (TEM) (TecnaiG220, Hillsboro, OR, USA) and the high-resolution transmission electron microscope (RHTEM) (JEM-2100, Tokyo, Japan) were used to observe the fine structure of the materials. Oxygen content was tested using an organ element analyzer (OEA) (Vario EL Cube, Frankfurt, Germany). The oxygen contained in the sample reacted with the carbon to form CO and CO_2_, and the amounts of these gases were measured to analyze the oxygen content of the Si powder sample.

## 3. Results and Discussion

**Figure 3:**
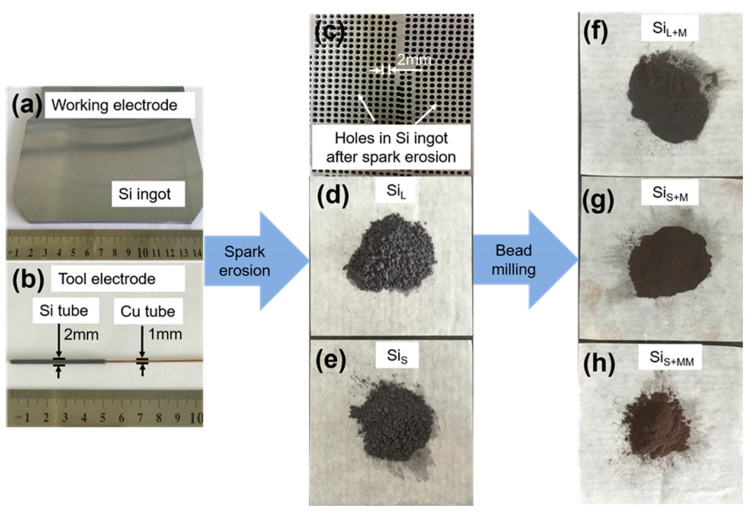
Pictures of (**a**) working electrode (Si ingot), (**b**) tool electrode (Si tube), (**c**) Si ingot after spark erosion, and powders of (**d**) Si_L_, (**e**) Si_S_, (**f**) Si_L+M_, (**g**) Si_S+MM_ and (**h**) Si_S+MM_.

Figure 3 shows the images of the original working electrode (Si ingot), working electrode (Si ingot), tool electrode (Si tube), Si ingot after spark erosion, Si_L_, Si_S_, Si_L+M_ and Si_S+MM_ powders. The tool electrode consisted of a Cu tube (the external and internal diameter was 1 mm and 0.5 mm, respectively) and a Si tube (the external and internal diameter was 2 mm and 1 mm, respectively). The Cu tube was used as an electrode conductor, and a liquid channel was inserted into the Si tube. With this structure, on one hand, copper tubes had better conductivity; on the other hand, silicon tubes were involved in spark erosion, and copper tubes will not vaporize or melt, thus avoiding the introduction of copper into the prepared silicon particles, the high purity of the material was maintained. After spark erosion, arrayed holes with a diameter of 2 mm were formed in Si ingot, which was controlled by automated tool positioning and servo system (Figure 1a). From Figure 3d–g, the colors of the Si particles changed from dark brown (Si_L_ and Si_S_) to tan (Si_L+M_, Si_S+M_ and Si_S+MM_), which was influenced by the size and shape of particles.

### 3.1. Particles Morphology

#### 3.1.1. Morphology of Si Micro/Nano Particles Prepared by Spark Erosion

**Figure 4:**
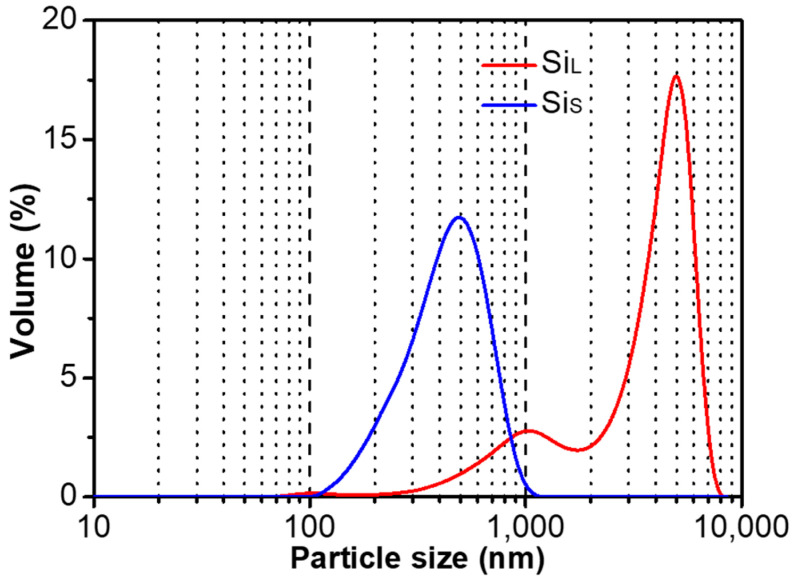
Particle size distribution of Si_L_ and Si_S_.

Figure 4 shows the particle size distribution of Si_L_ and Si_S_, and the abscissa is the logarithmic axis from 100 to 10,000 nm. The average particle size of Si_L_ and Si_S_ were 4060 nm and 390 nm, respectively. The average particle size and distribution of Si_L_ were much larger and wider than those of Si_S_. Increasing power density by reducing pulse duration raised the temperature of the plasma, which was in favor of melting/gasifying electrode materials forming smaller average particle size.

**Figure 5:**
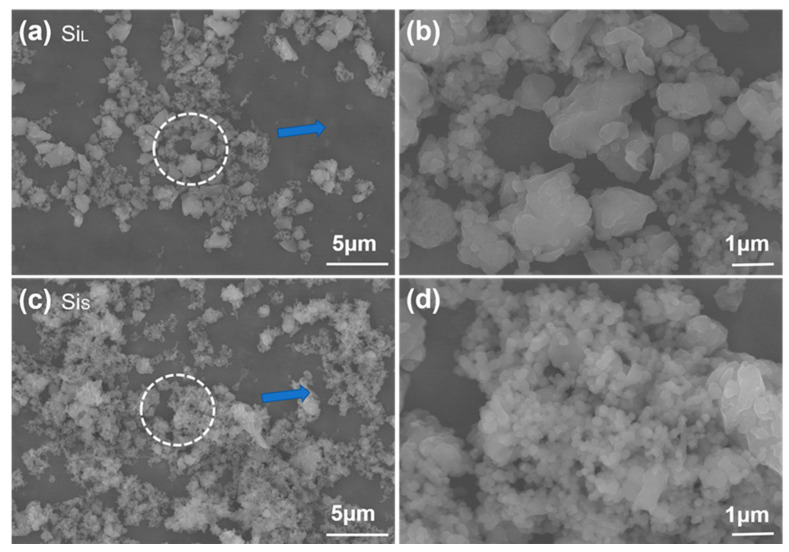
SEM images of (**a**,**b**) Si_L_ and (**c**,**d**) Si_S_.

SEM images of Si_L_ and Si_S_ in Figure 5a–d also display that the average particle size of Si_L_ is larger than that of Si_S_. Si_L_ had significant twin peaks on the particle size distribution curve (Figure 4), with significantly more micron-scale particles than submicron particles (Figure 5b), while Si_S_ had a large number of submicron and nanoscale particles (Figure 5d), which were more likely to be met by the gasification of silicon materials. When Si micro/nanoparticles were prepared by spark erosion in the central region of the plasma in Figure 1b, the silicon material vaporizes into atomic clusters in the region where the temperature exceeds the vaporization temperature. At the same time, in the regions where the temperature was lower but above the melting temperature of the silicon material, the silicon material melted to form sub-micron and micron-sized particles. Melted and vaporized Si particles rapidly cooled after encountering low-temperature working fluid and re-aggregated during the process of releasing energy [30].

**Figure 6:**
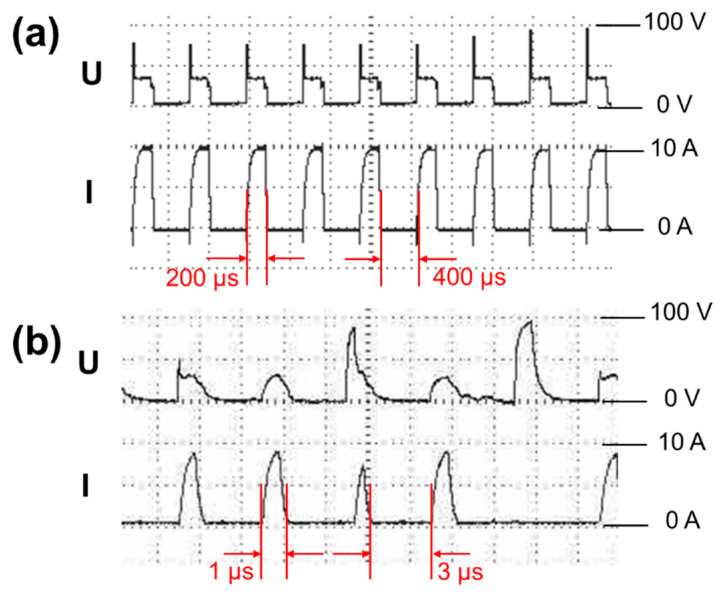
Discharge waveforms of (**a**) Si_L_ and (**b**) Si_S_ during spark erosion.

Figure 6 shows discharge waveforms of Si_L_ and Si_S_ during spark erosion. As the pulse duration of Si_S_ in Figure 6b was shorter, voltage waveform (U) and current waveform (I) of Si_S_ were irregular compared with those of Si_L_ (Figure 6a). The shorter the pulse duration was, the shorter the discharge time was, and the shorter the high-temperature lasted. Short high-temperature time will lead to the shrink of the melting area as well as the size of the formed molten particles; and finally, silicon micro/nanoparticles tended to agglomerate to release high surface energy. A long period of high-temperature caused many formed particles to agglomerate in the gasified and molten state, leading to the formation of covalent bonds between the particles (hard agglomeration). The agglomerated formed at low temperatures were soft agglomerates and easily dispersed. However, short pulse duration also decreased the yield of particles. The productivity of short pulse duration (1 μs) was around 4 g/h, and long pulse duration (200 μs) was about 35 g/h. The productivity of long pulse duration was higher because long high-temperature time expanded the melting area and formed plenty of melted particles.

#### 3.1.2. Morphology of Si Nanoparticles Prepared by Bead Milling

**Figure 7:**
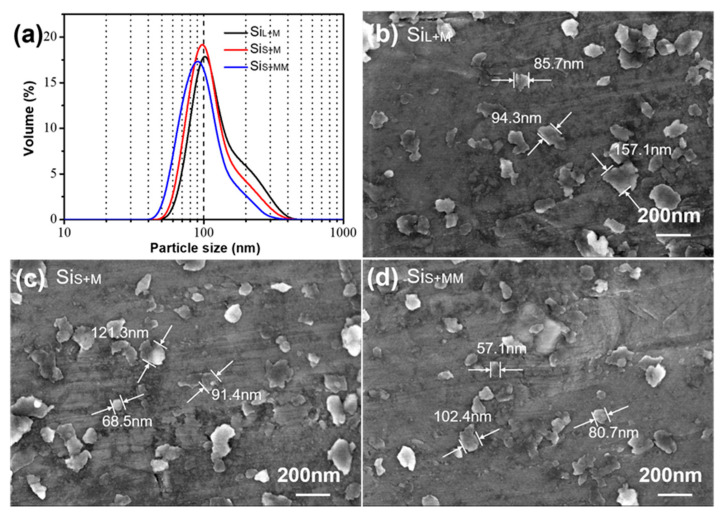
(**a**) Particle size distribution of Si_L+M_, Si_S+M_ and Si_S+MM_, SEM images of (**b**) Si_L+M_, (**c**) Si_S+M_ and (**d**) Si_S+MM_.

Monocrystalline silicon ingots are used to produce small particles by spark erosion which saves much grinding time for bead milling and greatly reduces energy consumption. Figure 7a shows the particle size distribution of Si_L+M_, Si_S+M_ and Si_S+MM_. Figure 7b–d exhibits SEM images of Si_L+M_, Si_S+M_ and Si_S+MM_, respectively. Si micro/nanoparticles (Si_L_ and Si_S_) were ground for 8 h under the same bead milling parameters obtaining Si_L+M_ and Si_S+M_. It can be seen from Figure 7 that particle size decreased obviously and had narrow distribution after bead milling. The average particle size of Si_S+M_ was 99 nm, smaller than Si_L+M_ (105 nm). Si_S_ was easier to be ground to the nanoscale than Si_L_. This was due to the larger particle size of Si_L_ and partly due to the existence of more hard agglomerations in Si_L_. Extending the bead milling time of Si_S_ to 12 h, the average particle size of Si_S+MM_ was 91 nm. Compared to Si_S+M_, the particle size of Si_S+MM_ was slightly smaller, which can be observed in Figure 7c,d as well.

Spark erosion and bead milling are based on internal energy and mechanical energy, respectively, to control particle size and internal structure. The mechanical energy of ZrO_2_ beads directly affects the normal and shear force generated during the collision, thus affecting the result of particle breakage. During the repeated collisions, the forces on the silicon particles will produce stress inside the particles. When the stress exceeds the limit that the particles can withstand, the particles will break into multiple particles. From Figure 5 and Figure 7, it can also be seen that the silicon particles formed after spark erosion were spherical in the majority, while the silicon particles formed after repeated collisions of ZrO_2_ beads in the process of bead milling continue to crack to form flake-like particles, which were much less than 100 nm in thickness. In this experiment, the productivity of nanoparticles was 25 g/h when the milling time was 8 h. The productivity of bead milling was determined by bead milling equipment, and sometimes, the production equipment could increase productivity dozens of times.

### 3.2. Internal Structure of Prepared Si Particles

The average crystallite size of Si particles was calculated by Scherrer formula from XRD patterns, and the results of Si_L_, Si_S_, Si_L+M_, Si_S+M_ and Si_S+MM_ are listed in Table 3 [34]. The Raman spectra of the 5 samples were analyzed by decomposing the spectra into the crystalline and amorphous bands. The Raman bands at near 510 cm^−1^ and 480 cm^−1^ were attributed to phonon modes of a-Si and c-Si, respectively, and the ratio of their integrated intensities could be used to estimate the amorphization degree because other bands at near 320, 430, 600 cm^−1^ were the LO, TA and combination modes, respectively, which was two orders smaller in magnitude than TO mode in the crystalline phase. Gaussian fitting was performed on the Raman peaks, near 480 cm^−1^ and near 510 cm^−1^. By calculating the integral intensity of curves, the amorphization degree estimated by Raman ($\rho_{a}$) was obtained from Equation (1) [35,36]:

(1) $$\rho_{a}=\left(1-\frac{I_{c}}{I_{c}+y^{c/a}I_{a}}\right)\times 100\%$$

where $I_{c}$ and$\;I_{c}$ are the integral intensity of a-Si and c-Si, respectively. $y^{c/a}$ is the ratio of Raman scattering cross-section of crystalline and amorphous components, and here, a typical value of $y^{c/a}=0.8$ is used. The analysis results of the 5 samples are shown in Table 3.

As a comparison, XRD pattern fitting was used to estimate amorphous ratio [37,38], and the curves of a-Si and c-Si at 28.4°, 47.3° and 56.1° were obtained, respectively. By calculating the integral intensity of curves, the amorphization degree estimated by XRD ($X_{a}$) was obtained from Equation (2):

(2) $$X_{a}=\left(1-\frac{I_{c}}{I_{c}+kI_{a}}\right)\times 100\%$$

where $I_{c}$ and$\;I_{c}$ are the integral intensity of a-Si and c-Si in XRD, respectively. k is a constant coefficient, and k=1 is usually used for elementary substances. The analysis results of the 5 samples are shown in Table 3. New Si particles reacted with H_2_O at high temperatures during spark erosion to form a SiO_2_ layer on the surface, and the fresh Si particles’ surface generated by collision during bead milling reacted with alcohol, forming oxides [39,40]. The oxygen contents of the 5 samples are presented in Table 3.

#### 3.2.1. Internal Structure of Si Micro/Nano Particles Prepared by Spark Erosion

**Figure 8:**
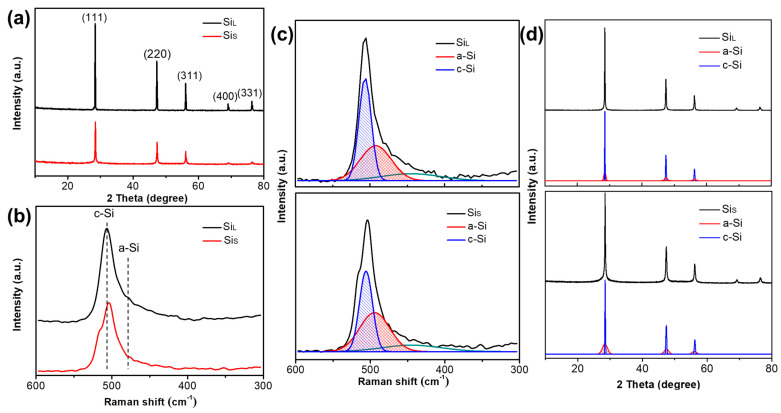
(**a**) X-ray diffraction patterns, (**b**) Raman spectrum, (**c**) Raman spectrum fitting and (**d**) XRD pattern fitting of Si_L_ and Si_S_.

The X-ray diffraction patterns and the Raman spectra of Si_L_ and Si_S_ are shown in Figure 8a–b, respectively. Diffraction peaks of Si_L_ and Si_S_ can be well indexed to the cubic Si phase (JCPDS. Card no. 01-0787) with (111) at 28.4°, (220) at 47.3°, (311) at 56.1°, (400) at 69.1° and (331) at 76.4°, as shown in Figure 8a. The intensity of the diffraction peak of Si_L_ was higher than that of Si_S_, and the peak width was narrower, indicating that Si_L_ had a higher crystallinity and a larger crystallite size, which was in agreement with the resulted from Raman spectra. The average crystallite sizes of Si_L_ and Si_S_ were calculated by Scherrer formula from XRD patterns to be 68.92 nm and 26.47 nm (Table 3).

As shown in Figure 8b, the peak at near 510 cm^−1^ corresponds to crystalline Si (c-Si), and the peak intensity of Si_L_ was obviously stronger than that of Si_S_, indicating that the degree of crystallization of Si_L_ was higher than Si_S_. A weak peak at near 480 cm^−1^ for both samples was assigned to amorphous Si (a-Si), indicating that the amorphous phase had already existed inside both samples. As shown in Figure 8c, Red curves were fitting curves of a-Si, and blue curves were those of c-Si. Curves in other colors in Figure 8c are peaks at other Raman shifts and the intensity, which was not used to calculate the amorphization degrees in Equation (1). By fitting the Gaussian peak of the Raman curve, combined with Equation (1), the amorphization degrees of Si_L_ and Si_S_ were 31.8% and 43.6%, respectively (Table 3). As shown in Figure 8d, the amorphization degrees of Si_L_ and Si_S_ were also estimated by fitting XRD patterns with Equation (2), and the results were 27.8% and 46.3% (Table 3), which agree well with those calculated by Raman.

**Figure 9:**
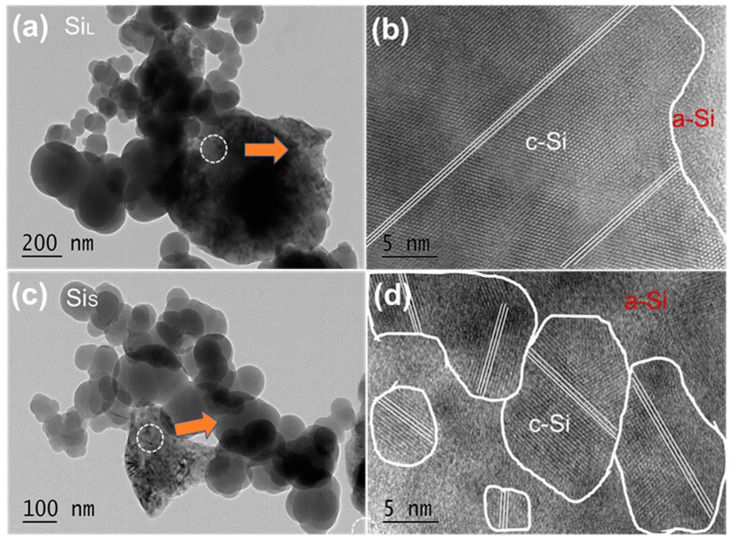
HRTEM images (**a**,**b**) of Si_L_ and (**c**,**d**) of Si_S_.

Figure 9 shows HRTEM images of Si_L_ and Si_S_. The nanocrystals are marked with a white circle, and the orientations are labeled by white lines. It can be verified from Figure 9b,d that the crystallite size of Si_S_ was smaller than that of Si_L_ and the crystal orientations were disordered in Si_S_.

The structure of SiO_2_ was amorphous, and the oxygen contents of Si_L_ (1.3%) and Si_S_ (2.5%) were both low (Table 3), which means that the dense SiO_2_ layer prevented the inner Si from being oxidized further. The oxygen content of Si_S_ was higher than that of Si_L_ because Si_S_ had a smaller particle size and, therefore, had a larger specific area than Si_L_. It could be deduced from these results that small pulse duration not only manufactures smaller particles but also created the smaller crystals embedded in a highly amorphous matrix.

During spark erosion, after vaporizing or melting, the silicon particles are either in a gaseous or a liquid state. Their internal structure changes from a single crystal structure to an amorphous structure, in which the silicon atoms are arranged irregularly. The amorphous structure contains higher potential energy, as the distance between the Si atoms is not equal to the distance in the equilibrium. During the condensation process, the amorphous structure recrystallizes in a short time and transforms into the crystalline structure that contains the smallest internal energy and is the most stable. The cooling rate has a very important influence on the size of crystallites [41,42]. Remaining at a high temperature for a long time will slow down the cooling rate of the formed particles, which will increase the time of the recrystallization process, resulting in forming a small number of crystal grains inside the particles with a larger size. As a result, the short pulse duration leads to short high-temperature time, which increases the cooling rate and the nucleation rate of the formed particles during recrystallization, as well as the number of crystal grains that have smaller size and higher disordered crystal orientations [43].

#### 3.2.2. Internal Structure of Si Nanoparticles Prepared by Bead Milling

**Figure 10:**
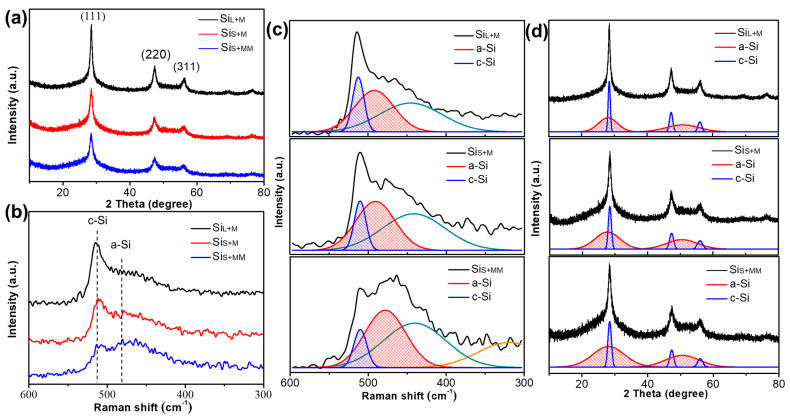
(**a**) X-ray diffraction patterns, (**b**) Raman spectrum, (**c**) Raman spectrum fitting and (**d**) XRD pattern fitting of Si_L+M_, Si_S+M_ and Si_S+MM_.

The X-ray diffraction patterns and the Raman spectrum of Si_L+M_, Si_S+M_ and Si_S+MM_ are shown in Figure 10. The average crystallite size of Si_L+M_, Si_S+M_ and Si_S+MM_ is calculated by the Scherer formula from XRD patterns being ~11.73 nm, 6.18 nm and 4.23 nm, respectively (Table 3). The peak intensity of Si_L+M_ was higher than that of Si_S+M_, but the peak width of Si_S+M_ was wider, indicating that the smaller the crystallite size of the particles before bead milling was, the smaller it was after bead milling. Comparing Si_S+M_ and Si_S+MM_ in Figure 10a, with the increase of the milling time, the intensity of the diffraction peak of Si_S+MM_ was further weakened, and the peak width was further broadened, indicating that increasing the milling time could further refine the grains and increase the amount of amorphous phase.

This conclusion is further verified in the Raman spectrum. As shown in Figure 10b, the c-Si peak of Si_L+M_ near 510 cm^−1^ was stronger than that Si_S+M_. Increasing the milling time, the c-Si peak of Si_S+MM_ becomes weaker and weaker. Simultaneously, the peak of a-Si near 480 cm^−1^ increased for all the samples with Si_S+MM_ > Si_S+M_ > Si_L+M_. According to Equation (1) and Figure 10c, the amorphization degrees ($\rho_{a}$) of Si_L+M_, Si_S+M_ and Si_S+MM_ were calculated to be 69.4%, 74.8% and 83.7%, respectively (Table 3). Similarly, the amorphization degrees ($X_{a}$) of these three samples estimated by Equation (2) and Figure 10d were 70.4%, 75.6% and 83.1% (Table 3). Through the analytical results of all 5 samples, we could conclude that the amorphization degrees estimated by fitting Raman spectra were highly similar to those calculated by fitting XRD patterns, which means the results are highly credible.

**Figure 11:**
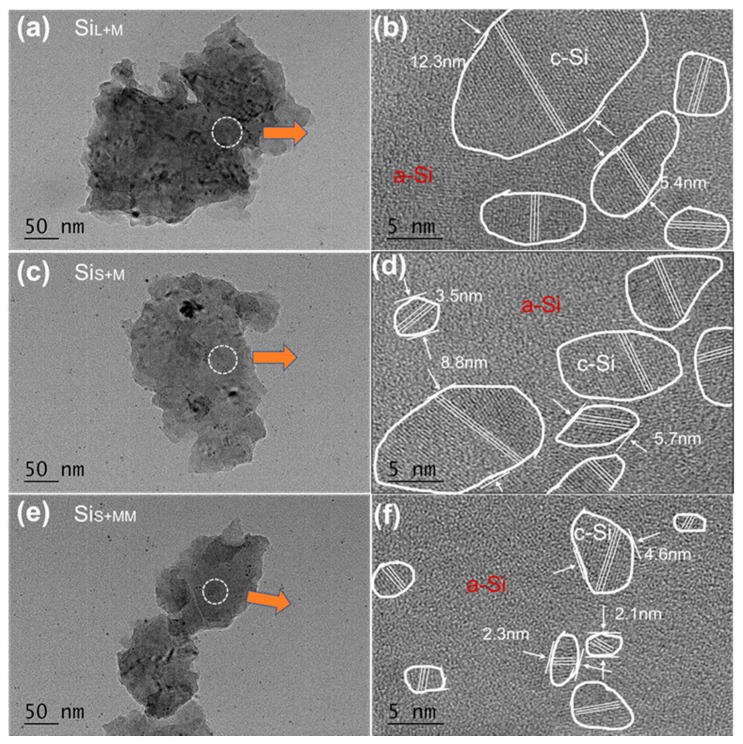
HRTEM images (**a**,**b**) of Si_L+M_ and (**c**,**d**) of Si_S+M_, and (**e**,**f**) of Si_S+MM_.

Figure 11 shows HRTEM images of Si_L+M_, Si_S+M_ and Si_S+MM_. The nanocrystalline structure embedded in the amorphous structure was formed inside. As a comparison to the raw material of high-energy ball milling and two-step bead milling (0.8, 0.1 mm ZrO2 beads), the internal structure of Si micro/nanoparticle was different and easier to form amorphous regions because these silicon particles were either in a gaseous or a liquid state during spark erosion [27,28]. For the same grinding time, Si_S+M_ had a smaller grain size and a higher degree of amorphization than Si_L+M_, which was mainly due to the difference in raw materials (Si_L_ and Si_S_); however, the difference between Si_L_ and Si_S_ had become smaller, indicating that bead milling for a long time could weaken the influence by raw materials. For the same raw material (Si_S_), Si_S+MM_ with longer bead milling time had a smaller grain size and a higher degree of amorphization than Si_S+M_, indicating that prolonging the grinding time could not significantly reduce the size of silicon particles, but it could further refine the crystal grains and form more amorphous structures.

However, the increase in grinding time also increased the degree of oxidation of silicon nanoparticles. The degree of oxidation evolved depending on the specific surface area of the particles and the bead milling time. The bead milling time of Si_L+M_ and Si_S+M_ was the same, but the increased oxygen content of Si_S+M_ (6.2%) was slightly higher than Si_L+M_ (5.8%) because of the smaller size of Si_S+M_. As the grinding time of Si_S+MM_ was 4 h longer than Si_S+M_, the oxygen content of Si_S+MM_ (8.7%) was obviously higher than Si_S+M_ (6.2%) (Table 3). Due to the repeated collisions of beads, when the receiving energy of a silicon atom in the grain of a silicon particle was greater than its bond energy, the distance between the atoms increased or decreased, and the potential energy increased. The atomic bond was broken to generate holes or lattice shifts, and the original parallel hexahedral structure was changed. The big grains were split into multiple smaller grains and even formed an amorphous structure with higher potential energy [33]. The greater the number of collisions, the smaller the crystallite size and the higher the proportion of the amorphous structure [44]. These effects were gradually transferred from the surface to the interior of the particles and gradually form a structure of Si nanocrystals embedded in amorphous Si.

This technical route combined the advantages of spark erosion and bead milling, e.g., spark erosion saved much time and energy for bead milling. From the observation of the morphology, internal structure and amorphization degree of Si particles, we could control the formation of nanocrystal and amorphous regions. Both spark erosion and bead milling produced defects inside the silicon particles, and bead milling could produce more defects than spark erosion because the bead milling takes a longer time and does not have a recrystallization process. Repeated collisions during the bead milling process resulted in the formation of a large number of point defects (vacancies, etc.), line defects (mixed dislocations), surface defects (grain boundaries, twin boundaries, etc.) in the particles, and the resultant Si particles were amorphous in a high degree. The processing parameters of spark erosion and bead milling had an important impact on the structure of resultant Si nanoparticles. The yield of this route to get Si nanoparticles depends more on spark erosion than on bead milling, as the yield of bead milling could be easily increased through the optimization of production equipment. The micro/nano Si particles could be collected as waste materials from the factory where Si bulk materials are processed by spark erosion, such as punching, slotting, etc.

## 4. Conclusions

In this study, a route combining two techniques (spark erosion followed by bead milling) was used to successfully prepare silicon nanoparticles that possess an amorphous structure with nanocrystals embedded. Short pulse duration was beneficial to decreasing particle size, refining the crystal grains, disordering the crystal orientation. However, spark erosion could only produce a small amount of amorphous phase. Bead milling further decreased the particle size, narrows the size distribution, refined the crystal grains and generated structures that were highly amorphous. Increasing the bead milling time added to these effects, but it also intensified the degree of oxidation of silicon particles. When the pulse duration was 1 μs and the milling time was 12 h, the average particle size and the average crystallite size of the prepared amorphous-like silicon nanoparticles reached 91 nm and 4.23 nm, respectively. These particles were in a high degree of amorphization (> 80%) and in low oxidation (< 10%). These physical properties of silicon nanoparticles could be controlled by adjusting processing parameters of spark erosion and bead milling.

## Figures and Tables

**Table 1:** The specific parameters of spark erosion.

Parameters	Si_L_	Si_S_
Working electrode	Si ingot (10 mm thick)	
Tool electrode	Si hollow tube (50 mm long, the external and internal diameter is 2 mm and 1 mm, respectively)	
Working liquid	Deionized water (resistivity is 10 MΩ·cm^−1^)	
Open-circuit voltage(V)	100	100
Peak current (A)	10	10
Pulse duration (μs)	200	1
Duty cycle	1:2	1:3

**Table 2:** The specific parameters of bead milling.

Parameters	Si_L+M_	Si_S+M_	Si_S+MM_
Grinding aid	Ethyl alcohol (1800 g)		
Beads	0.1 mm, 2 kg (ZrO_2_, bulk density is 3.5 kg/L)		
Effective volume of the bead milling equipment	0.7 L (filling rate of beads is about 81.6%)		
Linear velocity	13.5 m/s (rotation speed is 2300 r/min)		
Temperature of cooling water	<10 °C		
Raw material	Si_L_ (200 g)	Si_S_ (200 g)	Si_S_ (200 g)
Grinding time	8 h	8 h	12 h

**Table 3:** The average crystallite size, the amorphization degree and oxygen content of Si_L_, Si_S_, Si_L+M_, Si_S+M_ and Si_S+MM_.

Samples	The Average Crystallite Size (nm)	$\mathrm{The}\;\mathrm{Amorphization}\;\mathrm{Degree}\;\mathrm{by}\;\mathrm{Raman}\;(\;\rho_{a})\;(\%)$	$\mathrm{The}\;\mathrm{Amorphization}\;\mathrm{Degree}\;\mathrm{by}\;\mathrm{XRD}\;(X_{a})\;(\%)$	Oxygen Content (%)
Si_L_	68.92	31.8	27.8	1.3
Si_S_	26.47	47.5	46.3	2.5
Si_L+M_	11.73	69.4	70.4	5.8
Si_S+M_	6.18	74.8	75.6	6.2
Si_S+MM_	4.23	83.7	83.1	8.7

## Data Availability

The data presented in this study are available on request from the corresponding author.
